# Stabilized 30 µJ dissipative soliton resonance laser source at 1064 nm

**DOI:** 10.1038/s41598-024-76704-3

**Published:** 2024-11-16

**Authors:** Piotr Pokryszka, Yingchu Xu, Wonkeun Chang, Karol Krzempek

**Affiliations:** 1 Laser Spectroscopy Group, Faculty of Electronics, Photonics and Microsystems, Wroclaw University of Science and Technology, Wyb. Wyspianskiego 27, Wroclaw, 50-370 Poland; 2 School of Electrical and Electronic Engineering, Nanyang Technological University, 50 Nanyang Avenue, 639798 Singapore, Singapore

**Keywords:** Mode-locked fiber laser, Fiber amplifier, Dissipative soliton resonance, High energy pulses, Pulse duration stabilization, Output power stabilization, Lasers, LEDs and light sources, Nonlinear optics

## Abstract

We demonstrate the first successful stabilization of a dissipative soliton resonance (DSR) mode-locked (ML) laser source using straightforward techniques. Our setup employed a figure-8 (F8) resonator configuration and a nonlinear optical loop mirror (NOLM) to achieve stable mode-locking, generating 1064 nm rectangular pulses with a 3 ns duration at a repetition frequency of ~ 1 MHz. The pulses were boosted in an all-fiber amplifier chain and reached 30 µJ and 10 kW peak power per pulse at 30 W average output power. We addressed a critical gap in the literature by actively stabilizing key DSR pulse parameters: average output power (improved by a factor of 51), pulse repetition frequency (improved by 7583 using cross-phase modulation for synchronization), and pulse duration (improved by a factor of ~ 4). Additionally, we included a numerical analysis to explore the pulse formation mechanisms in DSR ML lasers working in a F8 configuration. Our findings show that non-complex all-in-fiber DSR ML lasers can reliably produce high-energy pulses with stable, repeatable parameters, making them suitable for future applications e.g. in nonlinear frequency conversion, laser micromachining, or LIDAR.

**Supplementary Information:**

The online version contains supplementary material available at 10.1038/s41598-024-76704-3.

## 1. Introduction

Fiber sources emitting ML laser pulses have been widely researched in recent decades. Active fibers doped with ytterbium or erbium ions have enabled the development of all-fiber laser configurations capable of generating short pulses in the near-infrared region^1,2^. Sources based on fibers doped with thulium or holmium allow using various ML techniques to achieve pulsed operation in the vicinity of 2 μm^3^. Extensive research has also been done on erbium and holmium-doped fibers, which provide gain in the mid-infrared (mid-IR) wavelength range. Soliton mode-locked (ML) fiber lasers have revolutionized the field of laser technology due to their compact size, excellent beam quality, and high efficiency^4^. However, due to the all-in-fiber design, the maximum extractable energy per pulse in a ML laser is limited by the interplay of several effects. Amongst those, the delicate balance requirement between the nonlinearities and the cavity dispersion - the soliton area theorem, is one of the main limitations in improving the output power of soliton all-fiber lasers^5^. Exceeding the gain boundary in a soliton ML laser in most cases results in pulse instabilities. The excess energy is commonly quantized into several pulses that coexist in the cavity. In such a case, harmonic ML, multipulse generation, soliton rain or soliton bunches are observed^6–9^. Achieving higher pulse energy requires external amplifier stages, with proper dispersion characteristics, thus is not always possible to set-up all-in fiber^10^.

In 2008, Akhmediev et al. published an article predicting that under specific cavity parameters, the energy of circulating ML laser pulses could in theory increase to infinite values^11^. Their calculations were based on the cubic-quintic Ginzburg-Landau equation (CGLE) and require six input parameters: cavity dispersion and attenuation, nonlinear amplification and its saturation, value of the high-order Kerr nonlinearity and bandwidth of the spectral filter. The results of their simulations suggested that with proper laser cavity design, a special arrangement of dissipative solitons could form in the cavity. This balance state would allow the pulses to maintain the ML characteristics and simultaneously accumulate significant amounts of energy. However, mitigating the limitations of the soliton area theorem comes at the expense of lengthening the pulse duration, usually to nanoseconds. Chang et al. further explored this effect, naming it Dissipative Soliton Resonance (DSR)^12^. Subsequent studies investigated that the DSR ML regime can be achieved in both net normal and anomalous dispersion cavities^13^. Inspired by these theoretical advancements, numerous laser configurations were demonstrated, ultimately confirming the possibility of generating high-energy pulses directly from the resonator, independently of the mechanism used to force the ML operation. Among others, DSR lasers employing the non-linear polarization evolution (NPE), figure-eight (F8) resonator configuration, figure-nine, non-linear optical loop mirror (NOLM), non-linear amplifying loop mirror (NALM) and saturable absorbers have been studied^14–27^. With proper cavity design, energies up to 12 µJ per pulse have been reported^28^.

Due to the requirement of reaching a certain value of cavity parameters (pulse repetition frequency, cavity dispersion, etc.), DSR ML lasers are usually built in all-in-fiber configurations, with resonators ranging from tens to hundreds of meters in length. Moreover, some take advantage of Double-Clad active fibers as the gain medium. Additionally, the distinctive feature of DSR ML lasers is the correlation between the cavity gain and the pulse duration. These characteristics result in significant drifts of fundamental parameters of the generated pulses: their duration, average output power and the pulse repetition frequency (e.g. due to optical pathlength perturbations and gain instability).

Despite the vast number of publications documenting DSR ML lasers, a critical gap remains in the published literature. Current research has primarily focused on exploring the influence of cavity dispersion on the laser operation, extracting high-energy pulses directly from the laser, or specific pulse properties. The current scientific literature lacks research on techniques that allow stabilizing the parameters of DSR pulses. This results in categorizing the DSR ML lasers merely as a temporary research area, without perspective for meaningful applications.

This paper aims to address this critical gap by exploring and combining several techniques to develop a DSR laser source capable of delivering ML pulses with high pulse energy and long-term stable parameters. This is the first demonstration of stabilizing the pulse parameters of a DSR ML laser. We experimentally verify methods for stabilizing the pulse duration, pulse repetition frequency and average output power of a DSR laser ML at ~ 1 μm. We believe that further development of DSR ML lasers with repeatable parameters will result in an outburst of their applications both in basic science and in out-of-lab applications.

Bullet points:

- Stabilized DSR ML Laser: Successfully demonstrated the first DSR ML laser source with crucial pulse parameters stabilized.
- Pulse Characteristics: Achieved 30 µJ pulses with 30 W average output power and 10 kW peak power.
- Stabilization Techniques: Implemented feedback loops improving average output power stability by a factor of 51, pulse repetition frequency by 7583, and pulse duration by ~ 4.

## Methods

### DSR laser configuration

**Fig. 1:**
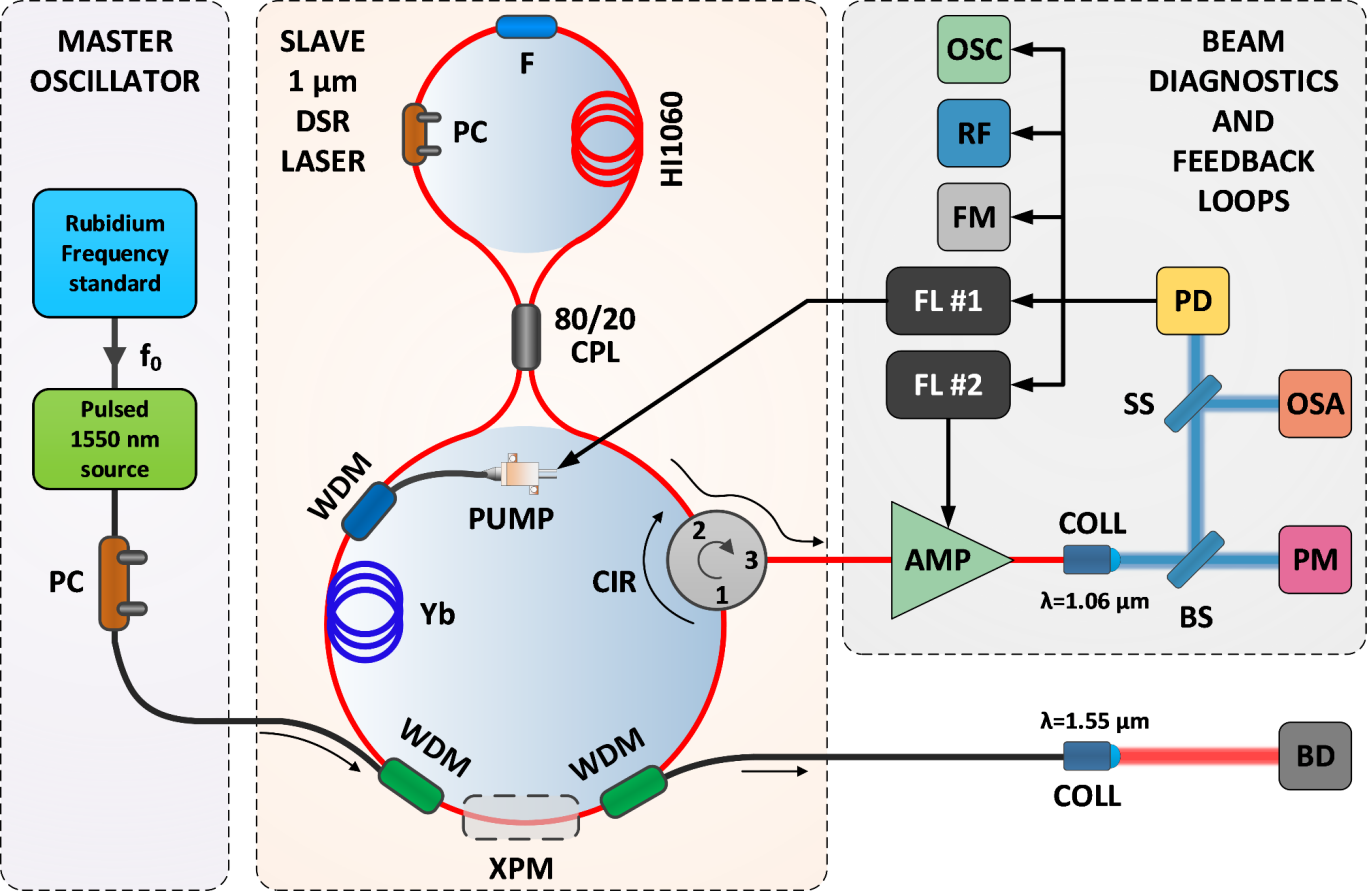
Schematic of the experimental setup. The setup was divided into three sections: the Master oscillator, the 1 μm DSR ML laser and the beam diagnostics and feedback loop section. F – filter, PC – polarization controller, HI1060 - spool of passive fiber, CPL – fiber coupler, CIR – fiber circulator, Yb - ytterbium doped active fiber, WDM – wavelength division multiplexer, PUMP – 976 nm pump laser, COLL – fiber collimator, FM – frequency counter, PM – power meter, OSC – oscilloscope, OSA, RF – optical and RF spectrum analyzers. PD – fast photodiode. AMP – fiber amplifier, BS – beam sampler, SS – beam splitter, BD – beam dump, FL – feedback loop. The area where the cross-phase modulation effect occurs is shown in the schematic.

The schematic of the experimental setup is provided in Fig. 1.

The DSR laser was built in a figure-8 (Fig-8) resonator configuration and a non-linear optical loop mirror (NOLM) was used as the ML mechanism^24^. A 165-meter-long spool of passive HI1060 single-mode fiber was added to the NOLM to achieve cavity parameters that favor DSR ML operation of the laser. A fiberized 2 nm bandpass filter (λ_center_ = 1063.5 nm) and a polarization controller (PC) were also placed in the NOLM. The NOLM was connected with the unidirectional loop using a 20/80 fiber coupler, which provided the necessary asymmetry in the counterpropagating pulses, and resulted in stable ML in the DSR regime. A fiber circulator was used in the unidirectional loop. This component acts as an isolator and simultaneously as an outcoupler for the generated pulses. The gain was provided by a 1.5-meter-long ytterbium doped (Yb) active fiber (SM-YSF-HI-HP, Coherent) optically pumped via a wavelength division multiplexer (WDM; blue on Fig. 1) with a 976 nm pump laser. Additional two WDMs designed for 1064 nm and 1550 nm wavelengths were spliced into the unidirectional loop of the laser. The WDMs (green on Fig. 1) were used to in-couple and out-couple 1550 nm Master pulses, which were used to synchronize the f_rep_ of the DSR laser (discussed in detail in Sect. 2.3). The laser begins to emit rectangular 2.2 ns ML pulses at a threshold pump power of 370 mW, after proper alignment of the polarization controller. The two paddles of the polarization controller were adjusted in approximately 5-degree increments, while carefully monitoring the signal from the fast photodiode on the oscilloscope for signs of characteristic DSR ML pulse formation. This iterative tuning process continued until rectangular-shaped pulses began to form. Once stable DSR ML operation was achieved, the polarization controller paddles were fixed in position. Notably, when the location of the cavity fibers remained unchanged between operation sessions, the DSR ML pulses were consistently reproduced at approximately the same paddle positions on the polarization controller. This tuning procedure ensures reproducibility of results under similar operational conditions.

**Fig. 2:**
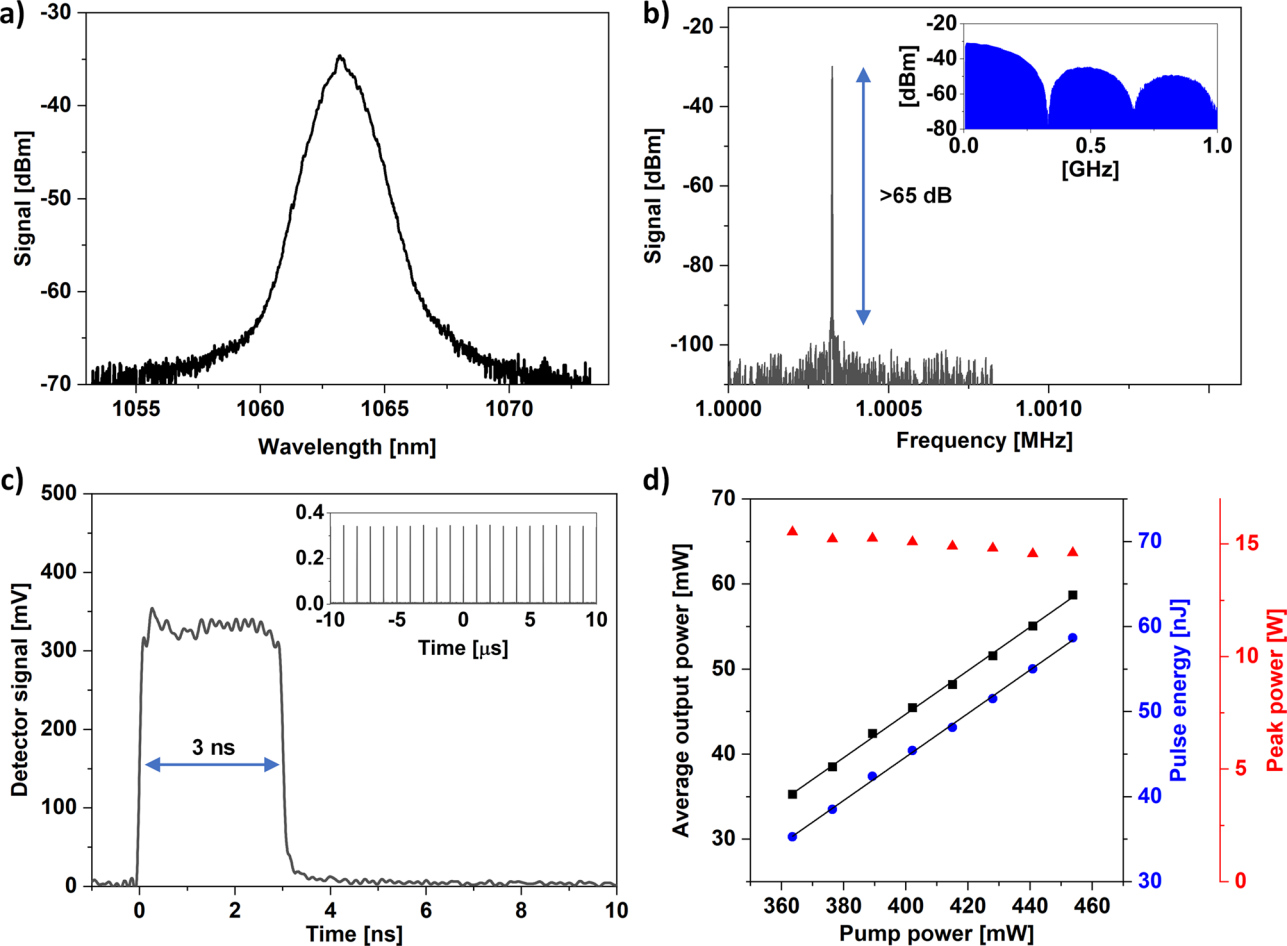
Optical (**a**) and RF spectrum (**b**) of the DSR pulses. Insets in (**b**) show the RF spectrum in a 1 GHz span. (**c**) 3 ns DSR pulse registered with a fast photodiode. The inset shows a train of 20 pulses. (**d**) crucial pulse parameters registered as a function of pump power.

The fundamental pulse repetition frequency (f_rep_) of the laser is f_rep_= 1.000321 MHz. As in a classical DSR ML laser, the pulse duration is directly correlated with the gain of the resonator, therefore, the pulse duration increases linearly with the coupled pump power, reaching 4 ns at 454 mW of pump power. Higher pump power results in pulse splitting, which was not further investigated in this research. At a pump power of 454 mW the laser was generating pulses with an average output power of 59 mW, which corresponds to a peak power of 14.5 W. During the following experiments, the DSR laser was set to generate 3 ns pulses. The optical spectrum, radio frequency (RF) spectrum, pulse shape and basic laser emission parameters are summarized in Fig. 2.

The center wavelength of the emission was 1064 nm. The sideband suppression ratio (SSR) measured with an RF spectrum analyzer reached 65 dB, which confirms the ML operation of the laser. The RF spectrum registered for a 1 GHz span shows a typical modulation, which is correlated with the pulse duration (in this case 3 ns). Similar RF spectrum modulation was observed in other experiments on both 1 μm and 1.55 μm DSR ML lasers^20,29–31^.

### Pulse amplification and average output power stabilization

**Fig. 3:**
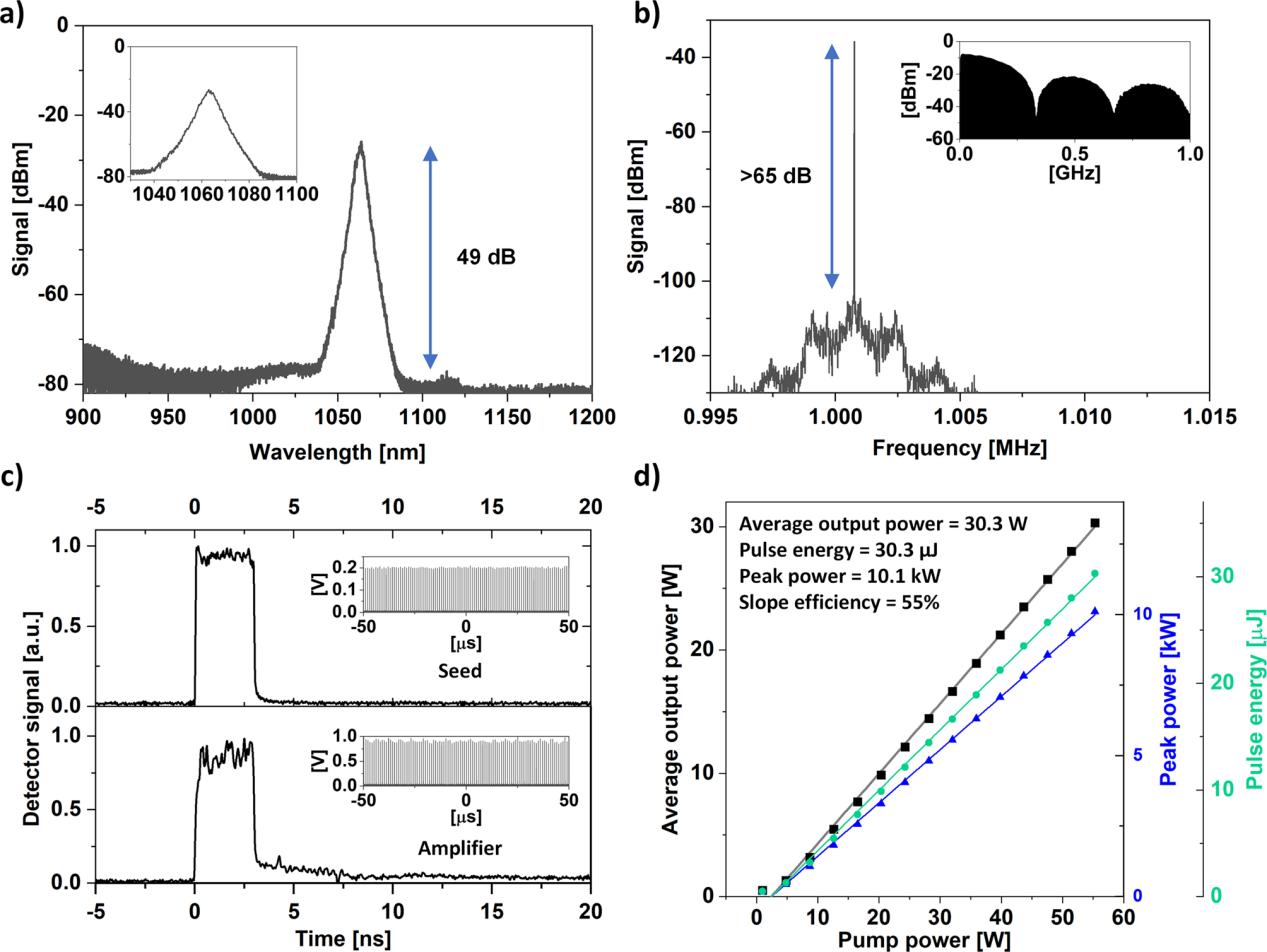
Pulse parameters after amplification measured at 30.3 W of average output power. Optical spectrum (**a**); inset shows a zoom. RF spectrum (**b**); inset shows the beatnotes registered for a 1 GHz span. Pulse shape (**c**); the DSR pulse is provided for comparison; 100 µs pulse trains are provided as insets. Crucial pulse parameters registered as a function of pump power delivered to the booster amplifier (**d**).

The DSR pulses were preamplified to 1.3 W and subsequently boosted to 30 W average output power in GTWave-based fiber amplifiers^32^. The pump lasers coupled to the pre-amplifier and the booster amplifier were controlled with a laser temperature and current drivers (ITC4020, Thorlabs). The amplified beam was out-coupled into free space and directed onto a beam sampler. This beam was used for diagnostics and to form feedback stabilization loops. The diagnostic beam was split with a beam splitter and directed to an optical signal analyzer and a fast photodiode (PD, Discovery semiconductors, DSC20H). The electrical signal from the photodiode was analyzed with an oscilloscope (DSO 90804 A, Agilent), RF spectrum analyzer (FSV3006, Rohde-Schwarz) and a frequency meter (Agilent, 53220 A). A set of pulse characteristics measured using the apparatus are plotted in Fig. 3.

After amplification, the optical spectrum of the DSR pulses broadens because of the high peak power. The signal-to-background ratio was 49 dB, showing no signs of amplified spontaneous emission build-up. Moreover, no residual pump was observed in the pulse spectrum, confirming that the output power measurements were not affected by unabsorbed pump light. The RF measurements show that the SSR did not deteriorate after amplification, nor does the 1 GHz span spectrum show any significant dissimilarities, when compared to the seed DSR laser. At a pump power of 55.4 W, the booster amplifier delivered rectangular shaped DSR pulses with 30.3 W of average output power. This corresponds to a pulse energy of 30.3 µJ and a peak power of 10.1 kW. The slope efficiency reached 55%.

It should be noted, that in this configuration the instabilities in the pulse parameters can originate separately from the DSR ML laser and the fiber amplifier. The pulse f_rep_ and pulse duration variations originate from the laser cavity itself, due to the sensitivity of the DSR regime to fluctuations in pump power and other environmental conditions (fiber temperature change, polarization change). As the pulses are relatively long, the nonlinear effects accumulated in the amplifier will have negligible time-varying influence on the pulse duration. In contrast, the average output power fluctuations can be contributed mostly to the last section of the high power amplifier chain, as the preamplifier and the booster amplifiers both work in saturation.

**Fig. 4:**
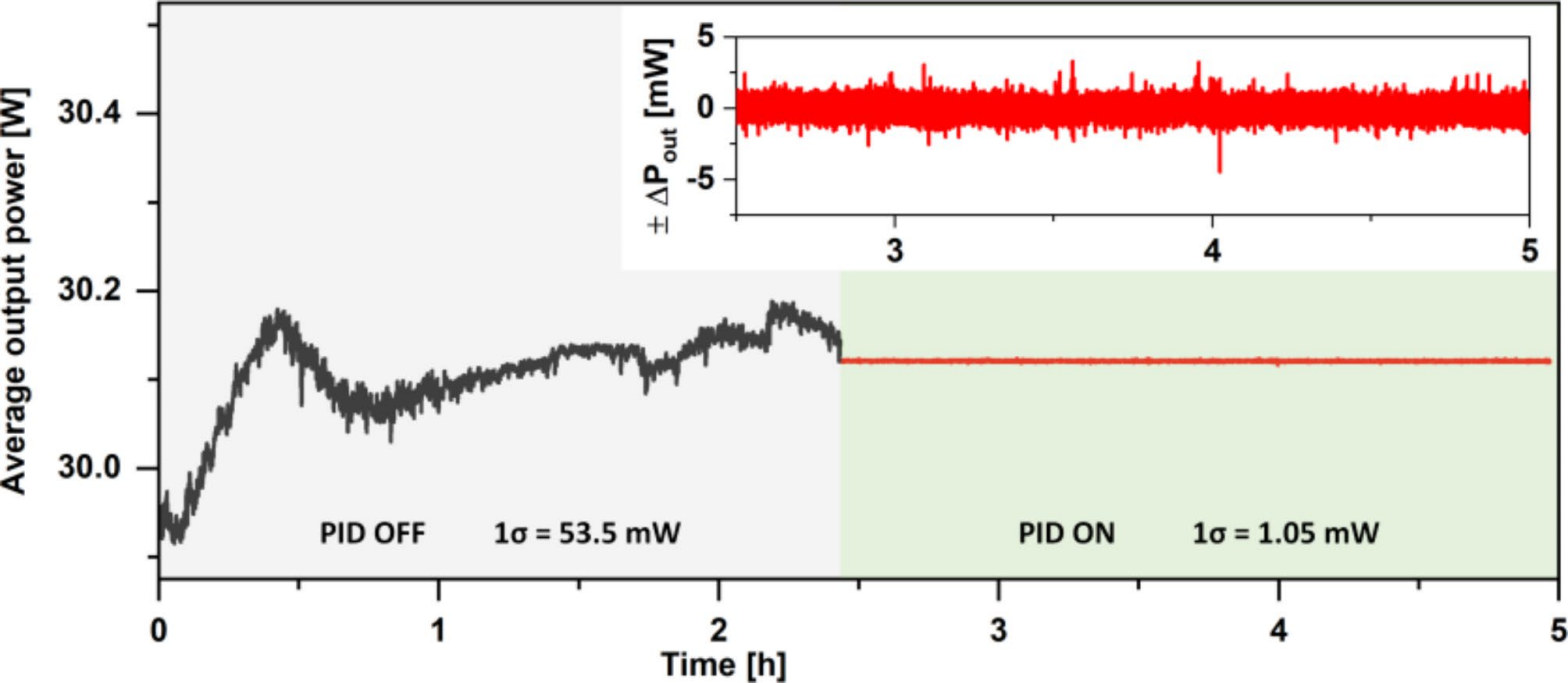
Results of the average output power stabilization. The inset shows a zoom in of the PID ON part of the graph, for clarity.

The fact that we used an external fiber amplifier allowed us to set up a noncomplex feedback loop, which was used to stabilize the average output power of the DSR laser source. For stabilization purposes, the electric signal from the photodiode (PD in Fig. 1) was coupled to a proportional-integral-derivative (PID) controller (D2-125, Vescent). We used a 2 kHz lowpass filter to monitor only the DC signal from the PD, which was used as the error signal for feedback loop #2 (FL #2). The correction signal was fed to the modulation input of the ITC4020 driver, which controlled the current delivered to the booster amplifier pump diodes. The performance of the average output power stabilization was measured in two steps. First, the DSR laser source was turned on and a 1 h thermalization period was allowed, to decouple any initial thermal drifts from the measurements. After this period, the average output power was measured for ~ 2.5 h. Next, the PID controller was turned on and set to stabilize the output power at 30.1 W. The measurement was repeated. The results of the experiment are plotted in Fig. 4.

Although the amplifier section was thermalized for 1 h, a significant drift in the average output power was observed during the 2.5 h measurement period. The 1σ standard deviation (SD) reached 53.5 mW, and the peak-to-peak drift was 312 mW. After engaging PID FL #2, the output power was stabilized at 30.1 W, with minimum drifts. The calculated 1σ SD was 1.05 mW for a measurement period of 2.5 h and the peak-to-peak drift did not exceed 6.7 mW. This is equal to a fractional instability of 3.49 × 10^−5^. Based on the measurements, we can conclude that the stability of the pulse average output power was improved by a factor of 51.

### Pulse repetition rate stabilization

The second parameter we stabilized in the DSR laser source was the pulse repetition frequency (f_rep_). Numerous methods have been developed to stabilize the f_rep_of pulse trains in ML lasers. Active locking techniques typically employ a piezoelectric transducer-based (PZT) fiber stretcher or a mirror mounted on a PZT, which have to be incorporated into the laser cavity^33,34^. However, these methods face limitations in the feedback bandwidth, stabilization dynamic range and long-term performance due to mechanical components and geometric constraints. An alternative approach focuses on stabilizing pulse f_rep_by controlling the refractive index (RI) of a fiber segment within the laser cavity. Typically, an auxiliary active fiber (doped fiber) is optically pumped with an additional pump laser, controlled by a feedback loop circuit. The resulting Kramers-Kronig-related RI change observed in the active fiber can be used to compensate for the laser cavity elongation. However, this approach is also restricted by its dynamic range and bandwidth (constrained by the lifetime of doped ion excited state) and typically necessitates initial thermal stabilization of the laser cavity for long-term synchronization^35–37^. Consequently, both techniques are unsuitable for DSR ML lasers, which in most cases have an order of magnitude longer cavity, compared to soliton ML lasers.

In our setup we have used an all-optical passive pulse repetition synchronization technique based on the cross-phase modulation (XPM) effect^38^. XPM is a nonlinear optical phenomenon resulting from the interaction between optical pulses within a medium, in our case between the Master oscillator pulses (provided from an auxiliary laser source; stabilized to a frequency reference) and Slave pulses (generated in the DSR laser cavity to be stabilized). The Master pulses are coupled into a part of the Slave laser cavity, where they induce a nonlinear RI variation via the Kerr effect, altering the phase of Slave pulses. Unlike PZT and pump-based stabilization techniques, utilizing a stable Master pulse source to induce XPM offers a high locking bandwidth (as the non-linear response of the medium can be nearly instantaneous) and excludes the need for setting up a feedback loop.

**Fig. 5:**
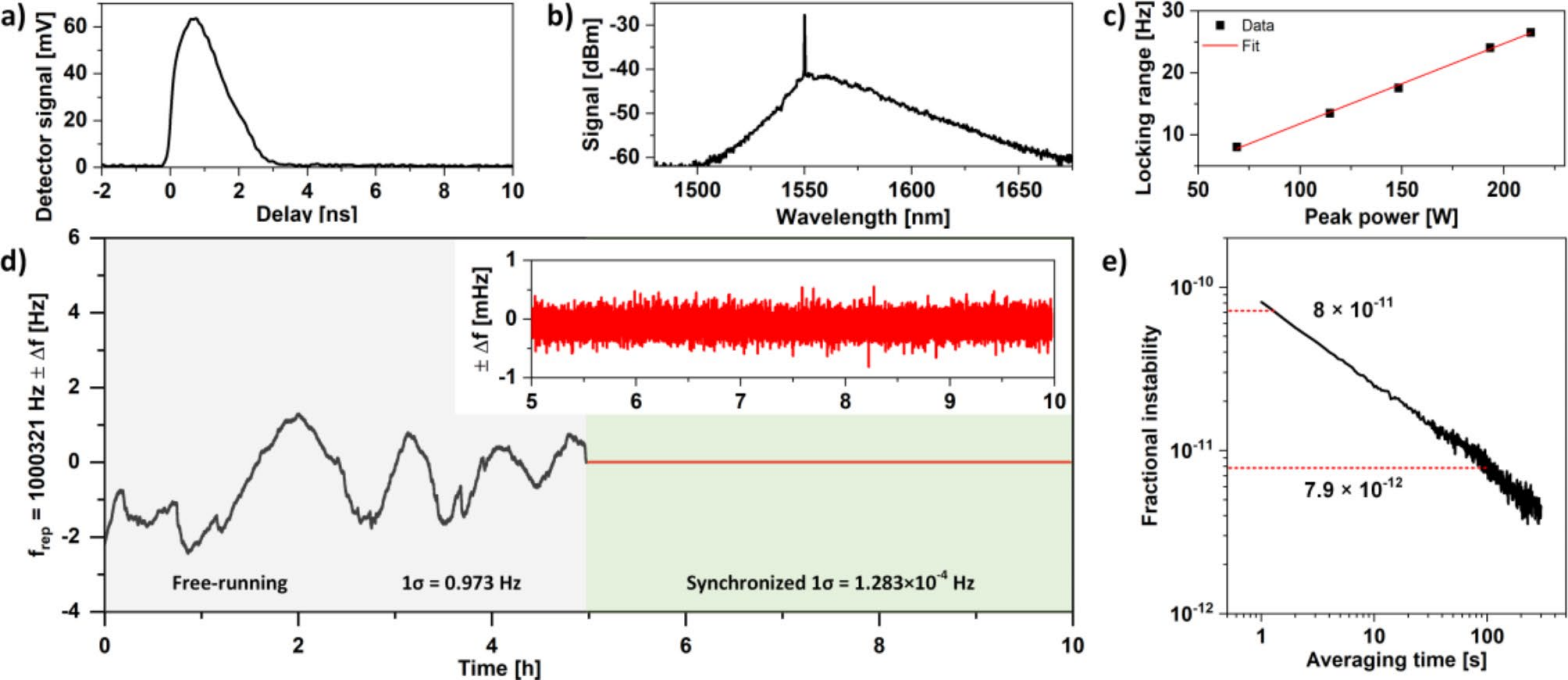
Master pulse shape (**a**) and spectrum (**b**). (**c**) synchronization range plotted as a function of the Master oscillator pulse peak power. (**d**) comparison of the free-running DSR laser f_rep_ deviations and during synchronization to the Master oscillator via the XPM effect. The inset shows a zoomed section of the synchronized plot part, for clarity. Master pulses with a peak power of 232 W were used during the measurements. (**e**) Allan deviation plot calculated based on data shown in figure (**d**) inset.

In our experiment, the XPM region was defined by splicing two WDMs in the unidirectional loop of the DSR laser (see Fig. 1). The Master oscillator utilized a 1550 nm distributed feedback laser (DFB) operating in the continuous wave (CW) regime with an output power of 12 mW. The beam was coupled to a semiconductor optical amplifier (SOA; SOA1117P, Thorlabs), mounted onto an SOA driver (Aerodiode). A function generator (33509B, Keysight) was used to trigger the SOA driver and generate a stable train of nanoseconds, 1550 nm pulses with a repetition rate matching the DSR f_rep_. We have used a frequency standard (FS; FS740, SRS) to provide a stable 10 MHz clock reference for the function generator and the frequency counter. The nanosecond pulses were amplified in an erbium-doped fiber amplifier and subsequently coupled into the DSR Slave laser cavity via a WDM. The Master pulses are extracted from the DSR cavity after the XPM region using a second WDM. During the experiments Master pulses with a peak power of 232 W were used. The Master pulse shape and optical spectrum is shown in Fig. 5a and b, respectively. To estimate the performance of the XPM-based f_rep_ stabilization we measured the free running frequency deviation for 5 h and compared it with the frequency deviation after injecting the Master pulses. The DSR laser was turned on for 1 h to allow initial thermalization of the DSR laser. The results are plotted in Fig. 5d.

A 10 Hz gate was used on the frequency counter when measuring the f_rep_ stability. The f_rep_ stability of the free-running DSR laser reached a 1σ SD of 0.973 Hz and a peak-to-peak deviation of 4.15 Hz, during a 5 h measurement. After synchronizing the DSR laser with the Master oscillator, the measured 1σ SD reached 1.283 × 10^−4^ Hz. This corresponds to an improvement factor of 7583. In addition, the measured f_rep_ stability is similar to the performance of the generator used to generate the Master pulses (1.116 × 10^−4^). The fractional instability of the DSR ML laser pulses after synchronization with the rubidium clock reached 1.282 × 10^–10,^ for a 10 Hz gate. Based on the calculated Allan deviation (shown in Fig. 5e, the fractional instability of the DSR f_rep_ reached 8 × 10^–11^ and 7.9 × 10^–12^, for 1s and 100 s integration time.

The dynamic range of the XPM synchronization of the pulse repetition rate in a Master-Slave configuration is heavily influenced by the Master pulse duration, as the XPM effect is dependent on the peak power of the injected pulses. In our setup, the peak power was limited by the minimum pulse duration achievable by the SOA-based seed source, approximately 1.75 ns (full-width at half-maximum), and by the extractable power from the fiber amplifier chain. While shorter pulses could have provided higher peak power and higher synchronization dynamic range, achieving such pulses would require employing more expensive components into the system, like electro-optical amplitude modulators and fast generators. These additions would significantly increase system costs and complexity, which we aimed to avoid. As shown in Fig. 5c and d, the synchronization dynamic range achieved with the SOA-based Maser source exceeded the free-running instabilities by nearly an order of magnitude, thus was sufficient for its application.

### Pulse duration stabilization

The stability of laser operating regimes and polarization orientations, as well as average power stabilization systems, have received substantial attention to date^39–42^. Nevertheless, the study of pulse duration (width) stabilization has received far less attention. In spite of this, pulse duration stability is essential for numerous applications. This emphasizes the necessity of having an efficient and reliable method for monitoring and controlling this parameter. Currently, many of the most widely used techniques require elaborate mathematical retrieval processes or complex setups^43^. For pulse duration measurements, nonlinear approaches have been widely used, however, these methods have substantial drawbacks^44–46^. Additional expensive parts, like nonlinear crystals and high-bandwidth photodetectors able to parametrize the downconverted pulses, are needed for the nonlinear optics-based method. Moreover, the majority of nonlinear techniques necessitate accurate calibration, which further complicates the measurement procedure.

**Fig. 6:**
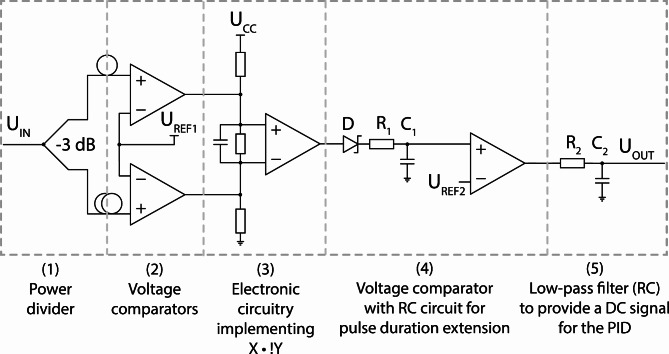
Circuit diagram of the pulse stretcher. I_IN_/U_OUT_ – input/output voltage, U_CC_ – supply voltage, U_REF1,2_ – reference voltage, D – Schottky diode, R_1,2_ – resistor, C_1,2_ – capacitor.

**Fig. 7:**
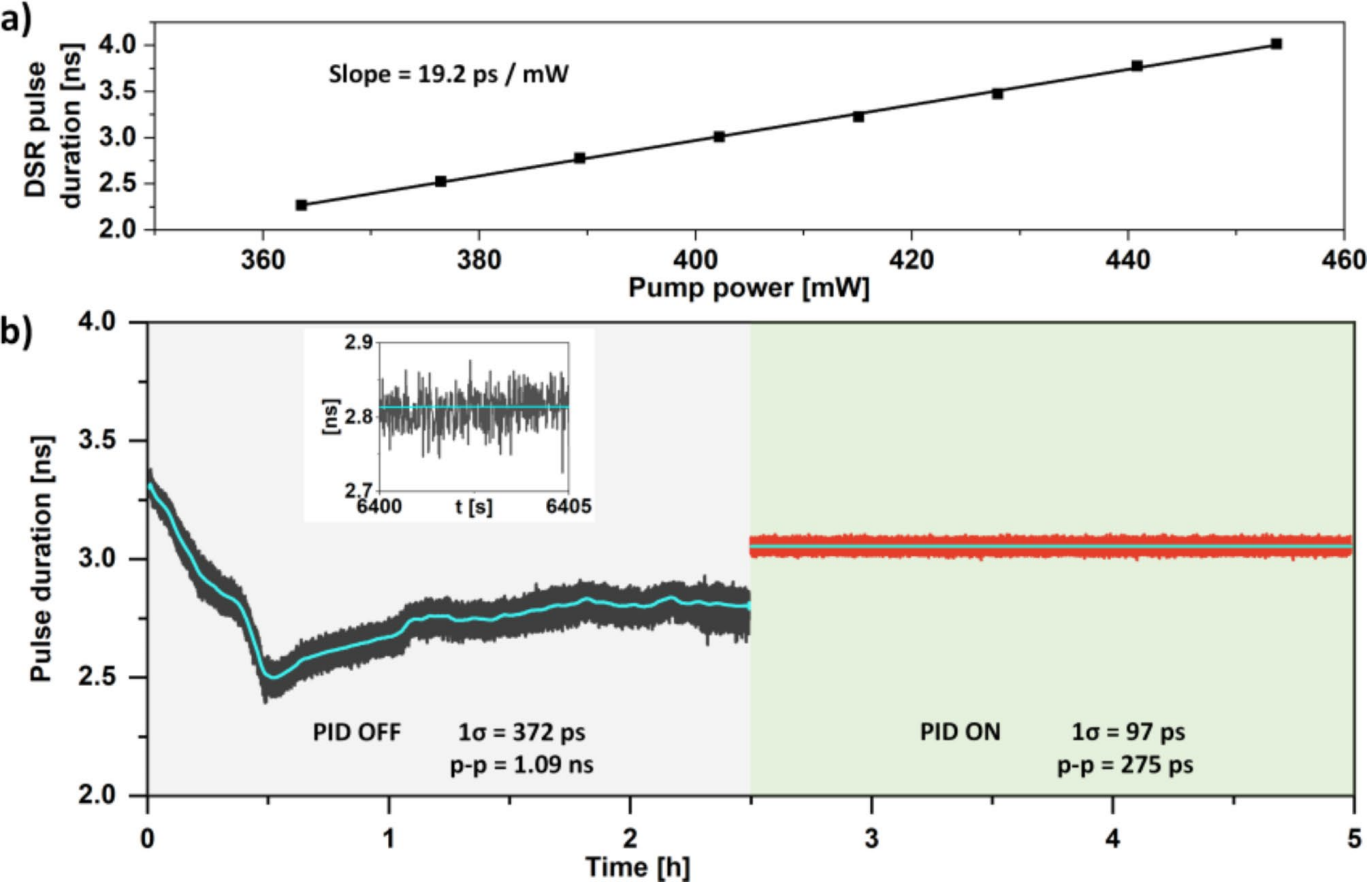
DSR pulse duration measured as a function of pump power  (**a**), comparison of the free-running pulse duration deviations and during stabilization (**b**). Inset in figure (**b**) shows a zoom of the PID OFF part of the graph, for clarity. The blue line in the graphs shows the data trend.

DSR lasers in most cases generate pulses with nanosecond durations. This allows to employ direct and non-complex approaches to determine the time-varying duration of the pulses, which can be subsequently utilized to build a feedback loop. The pulse duration of DSR ML lasers is correlated with the gain of the resonator^15,22,30^. In our setup, the gain was controlled by varying the current of the pump laser. The correlation between pump power and the DSR pulse duration is plotted in Fig. 7a. This distinguishing feature has been utilized by us to set-up the FL #1, to stabilize the pulse duration. The FL #1 consisted of a custom electronic circuit and a PID controller (D2-125, Vescent). The electronic circuit is based on a logarithmic pulse stretcher (LPS) and converts the pulse duration into a voltage signal interpretable by the PID. The LPS utilizes a resistor–capacitor circuit (RC) for the pulse stretching functionality and is depicted in schematically in Fig. 6.

The voltage in the circuit decays logarithmically with a discharge time constant (τ) that depends on the parameters of the R and C elements. The presented electronic circuit employs capacitors with C0G characteristics (temperature compensating) and a resistor manufactured using thin-film technology, which exhibit high temperature stability and high manufacturing precision. The LPS circuit employs an Analog Devices LTC6752 voltage comparator, characterized by a rise and fall time (t_r_, t_f_) of 1.25 ns for a supply voltage of 5 V. During the design of the electronic circuit, a key electronic component was identified as the RC circuit’s discharge direction blocking diode, or rather, its capability to repolarize the junction from the conduction direction to the reverse direction. This parameter defines the shortest possible pulse duration that can be propagated through the diode. For general-purpose Schottky diodes, the time required to repolarize the junction from the conduction direction to the reverse direction ranges from single to tens of nanoseconds. The LPS presented here uses a specialized Schottky diode (BAS81-GS08, Vishay Semiconductors) having a junction capacitance of C = 1.6 pF and allowing operation with pulses shorter than 1 ns. The Logarithmic Pulse Stretcher can be divided into five functional blocks. The first block (1) has divides the power into the branches and delays the propagation through one of them. In our circuit, the relative delay between the pulses in each branch was realized by using a RG-58 coaxial cable with the length adjusted experimentally. The second block (2) was constructed based on a pair of comparators designed to increase the amplitude of the signal and generate a pulse with steep slopes. The third block (3) implements a logic operation X·!Y, which is used to detect the delay of signal X relative to signal Y. The penultimate (4) is an R_1_C_1_ circuit that includes a blocking diode and a voltage comparator. We have added a low-pass filter (R_2_C_2_) in block (5) to convert the stretched pulses into a DC signal, which was required as an error input of the PID controller. The value of the DC signal is proportional to the length of pulses generated by the LPS circuit. The correction signal was coupled to the modulation input of the laser driver that controls the pump power delivered to the DSR ML laser cavity. The performance of the chosen stabilization technique was estimated based on two measurements. First, the pulse duration deviation was recorded for 2.5 h under free-running conditions. Next, the measurement was repeated with FL #1 turned ON. The pulse duration was measured with a fast photodiode (Discovery semiconductors, DSC20H). The electrical signal from the photodiode was analyzed with an oscilloscope (DSO 90804 A, Agilent), and the pulse duration values were extracted to a LabView application. The results are plotted in Fig. 7b.

A slope of 19.2 ps per mW of pump power was measured, based on the results in Fig. 7a. This shows that the distinctive feature of the DSR ML lasers can be used to stabilize the pulse duration with significant precision. For a free-running case, the 1σ SD of the pulse duration reached 372 ps, with a peak-to-peak drift of 1.09 ns. The inset shows that the pulse duration deviates several times per second. After engaging the FL #1, the pulse duration was stabilized at a value of ~ 3 ns during a 2.5 h measurement period. The 1σ SD reached 97 ps and the peak-to-peak drift did not exceed 275 ps. The measurements show that an inexpensive and noncomplex electronic circuit can yield a pulse duration stability improvement factor of ~ 4 for the peak-to-peak drift and the 1σ SD. The long-term pulse duration drift was eliminated, however, further reduction of the subnanosecond deviations will require a circuit with higher bandwidth. We believe that a significant improvement could be achieved if a field programmable gate array (FPGA) electronic circuit were used for pulse duration measurement and error signal generation. Further experiments will focus on investigating this approach.

## Dissipative soliton resonance pulse formation theory and simulations

**Fig. 8:**
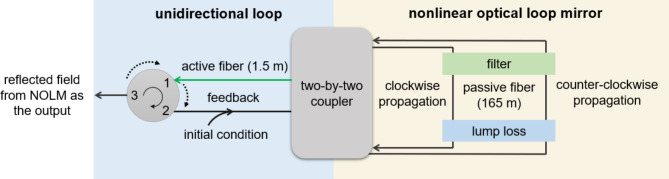
Schematic of the numerical model.

A dissipative soliton forms due to a delicate balance between gain, loss, dispersion, and nonlinearity^47^. Specifically, in an all-normal dispersion laser cavity, the soliton forms between pulse broadening, caused by the combined effects of normal dispersion and self-phase modulation, and pulse compression, facilitated by spectral filtering^48^. We studied the dissipative soliton dynamics in this laser cavity numerically. As shown in Fig. 8, a model comprising a chain of equations describing the effects of components comprising the cavity was solved. The numerical model consists of a nonlinear optical loop mirror (NOLM) allowing bidirectional propagation and a unidirectional loop. In the NOLM, the initial condition and subsequent circulating field are injected into one of the input ports of an 80:20 two-by-two coupler. The coupler splits the input field into two counter-propagating fields, described as:

1 $$\:{A}_{\text{cw}}=i\sqrt{1-k}\cdot\:{A}_{in}$$

2 $$\:{A}_{\text{ccw}}=\sqrt{k}\bullet\:{A}_{in}$$

where *A*_in_ is the input field, *A*_cw_ denotes the clockwise propagating field, *A*_ccw_ describes the counterclockwise propagating field, *k* is the coupling ratio which is 0.8 in this case. The clockwise propagating field passes through the bandpass filter with a center frequency of 1063.5 nm and a full width at half maximum (FWHM) bandwidth of 2 nm. After the filter, the field passes through a 165 m-long passive fiber after which it experiences a 1.5 dB lump loss and is guided to another output port of the coupler. Meanwhile, the counterclockwise propagating field is simulated similarly, except that it encounters the lump loss first, followed by the passive fiber and the filter. The two fields are recombined in the coupler. The transmitted field, *A*_t_, and the reflected field, *A*_r_ from the NOLM are calculated as:

3 $$\:{A}_{\text{t}}=i\sqrt{1-k}\bullet\:{A}_{cw}+\sqrt{k}\bullet\:{A}_{ccw}$$

4 $$\:{A}_{\text{r}}=\sqrt{k}\bullet\:{A}_{cw}+i\sqrt{1-k}\bullet\:{A}_{ccw}$$

The transmitted field enters the unidirectional loop and propagates through a 1.5 m-long active fiber. A circulator is placed in the unidirectional loop to outcouple the reflected field from the NOLM while the transmitted field is fed back to the input port of the coupler for the next roundtrip.

When a short pulse propagates through the active fiber, it evolves due to various effects, including group-velocity dispersion, self-phase modulation, attenuation, and gain. To describe the evolution of the complex pulse envelope in the active fiber, a modified nonlinear Schrödinger equation was solved:

5 $$\:\frac{\partial\:A}{\partial\:z}=-\frac{\alpha\:}{2}A-i\frac{{\beta\:}_{2}}{2}\frac{{\partial\:}^{2}A}{\partial\:{t}^{2}}+i\gamma\:{\left|A\right|}^{2}A+\frac{{g}_{0}/2}{1+{\int\:}_{-{\infty\:}}^{{\infty\:}}\left|A\right|dt/{E}_{\text{sat}}}\left(A+\sigma\:\frac{{\partial\:}^{2}A}{\partial\:{t}^{2}}\right)$$

where *A*(*t*,*z*) is the complex envelope, *t* is the timeframe moving at the group velocity, and *z* is the propagation length. *α* denotes the intensity attenuation coefficient, *β*_2_ is the second-order dispersion parameter, and *γ* is the nonlinear coefficient describing the self-phase modulation. Three parameters define the gain, i.e., *g*_0_, *E*_sat_, and *σ*, which represent the small-signal gain, gain saturation energy, and gain bandwidth, respectively. The propagation in the passive fiber in the NOLM can be modeled by the same equation with different fiber parameter values and without the gain term. We have summarized the simulation parameters in Table 1.

**Table 1:** Simulation parameters that form a stable dissipative soliton with similar properties to the experimental observations.

Parameter	Value
*β*_2,active_	22.8 ps^2^ km^−1^
*γ*_active_	3.5^−1^ km^−1^
*g*_0_	2 m^−1^
*E*_sat_	8 nJ
*σ*	0.05 ps^2^
*β*_2,passive_	21.8 ps^2^ km^−1^
*γ*_passive_	5 W^−1^ km^−1^

**Fig. 9:**
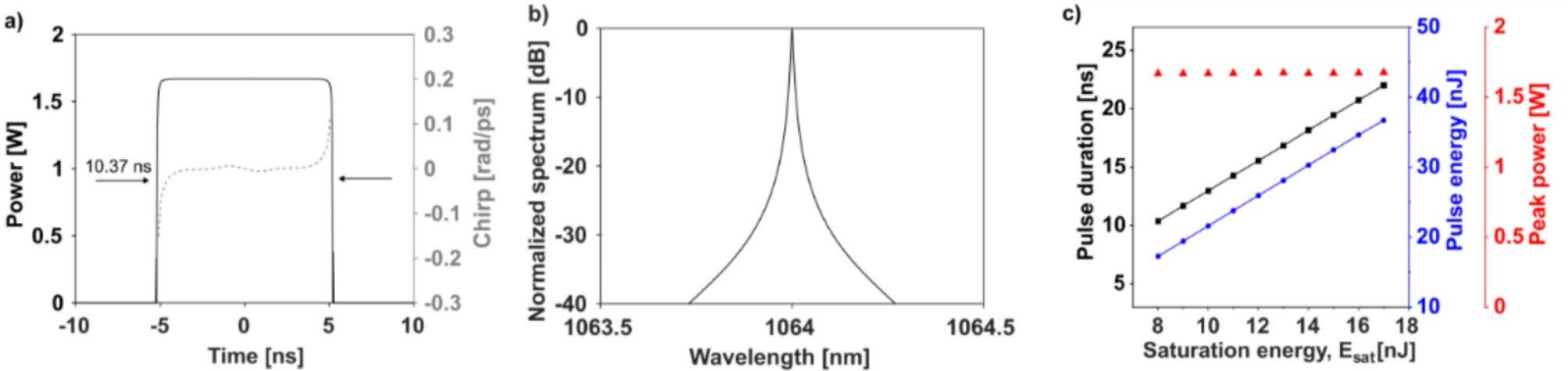
(**a**) A stable dissipative soliton corresponding to system parameters in Table 1. (**b**) Its associated spectrum. (**c**) Pulse duration, pulse energy, and peak power with different levels of *E*_sat_.

While we were able to obtain most of the simulation parameters directly from the component datasheets, the three gain parameters could only be determined through trial and error. Table 1 shows a set of parameters that results in the formation of a stable pulse shown in Fig. 9a. Its spectrum is presented in Fig. 9b. The pulse has an FWHM duration of 10.37 ns, energy of 17.26 nJ, and peak power of 1.67 W. The simulated pulse in Fig. 9a lacks the ripples on the pulse top observed experimentally in Figs. 2c and 3c. These ripples are a manifestation of the complex interplay between nonlinearities in the fiber and gain medium, which also show up during the transient phase in our simulations. They decay in the simulation as the solution converges after many roundtrips.

Using the pulse shown in Fig. 9a and b as an initial condition, we studied the system response to increasing pump power. This is simulated by increasing *E*_sat_ in Eq. (1)^49^. Figure 9c shows the plots of the pulse duration, energy, and peak power as a function of *E*_sat_. Both the pulse duration and energy increase linearly with *E*_sat_​ from 10.37 to 22.01 ns and from 17.26 to 36.68 nJ, respectively. On the other hand, the peak power does not change significantly around 1.67 W. These features closely resemble the observations reported when the laser operates in the DSR regime^12^.

While the qualitative features of the laser dynamics observed in the experiments are closely represented in our numerical model, there are some quantitative discrepancies. We suspect the differences are due to the simplified gain saturation model, where the time-dependent gain saturation is not accounted for. The influence of the time-dependent gain saturation is not negligible when a long pulse circulates in a fully saturated regime^50^. The leading edge of the pulse would experience higher gain, while the trailing edge would be suppressed due to saturated gain, resulting in a shorter pulse duration and broader spectral bandwidth.

## Discission and conclusions

In this paper, we demonstrated the first successful stabilization of a DSR ML laser source using simple yet effective techniques. Our experimental setup, which incorporated a figure-8 resonator configuration and a nonlinear optical loop mirror (NOLM), enabled stable mode-locking, generating 1064 nm rectangular 3 ns pulses at a fundamental repetition frequency of 1.000321 MHz. The DSR pulses were subsequently amplified in an all-fiber booster, and reached 30 µJ and 10 kW per pulse at an average output power of 30 W.

Our work significantly advances the field of high power pulsed sources by addressing the critical gap in stabilizing key pulse parameters of DSR ML lasers. We have shown that the use of feedback loops for stabilizing average output power and pulse duration, as well as the application of cross-phase modulation for pulse repetition frequency stabilization, can greatly enhance the long-term stability of these laser systems. These findings open new possibilities for the deployment of DSR ML lasers in practical applications that demand consistent high-energy pulses, such as nonlinear frequency conversion, laser micromachining, or LIDAR.

The practical advantages of our approach are evident in the stability improvements achieved. We have used the pulse amplification stage to implement a feedback loop, which allowed for the stabilization of the average output power, significantly reducing drift, and enhancing stability by a factor of 51. Moreover, we introduced a unique approach for frep stabilization based on the XPM effect, enabling synchronization of the DSR pulse trains to a stable Master oscillator. Using XPM induced by stable Master pulses, we achieved a remarkable improvement factor of 7583 in the frep stability. Additionally, we addressed pulse duration stabilization using the distinctive correlation between pulse duration and resonator gain in DSR ML lasers. Our simple feedback loop implementation improved the pulse duration drift by a factor of ~ 4. The techniques we employed are straightforward and eliminate the need for complex or mechanically moving components within the laser cavity, making our method both cost-effective and scalable for various industrial applications.

Apart of the experimental results, we additionally included a numerical section into our article. This allowed us to study the mechanism governing the complex pulse formation in DSR ML lasers. Moreover, this section includes a description of the methods we have used to simulate the constructed pulse laser source, along with all crucial parameters required to reproduce the calculations.

Despite these advances, our study has limitations that should be acknowledged. While we achieved significant stability improvements for pulse prepetition frequency and average output power, further enhancements in pulse duration stabilization may require more sophisticated electronics, such as FPGA-based systems. Although significantly more expensive, the FPGA-based electronics should result in a significant reduction of the long-term pulse duration drifts.

Building on our results, future research could explore the development of more advanced feedback systems, potentially, as discussed above, incorporating FPGA technology to further enhance the pulse duration stability. Additionally, expanding the range of laser configurations tested with these stabilization techniques would provide a broader understanding of their applicability. Finally, investigating the integration of these stabilized DSR ML lasers into complex optical systems, e.g. in precision laser micro-machining or nonlinear frequency conversion applications would provide significant insight into the performance of the developed stabilization techniques.

While the achieved 30 µJ pulse energy in our DSR laser system is lower than the pulse energies exceeding 1 mJ reported in other pulsed amplified systems^51^, it is important to emphasize the specific context and objectives of our work. Although we utilized external amplifiers, the primary focus was on maintaining pulse stability within the DSR regime while achieving relatively high pulse energy. The 30 µJ pulses generated in our system, while below the maximum reported values, represent a balanced optimization of pulse energy and stability, which is crucial for various applications.

In the supplementary materials, we have provided detailed information on the electronic circuit used for pulse duration stabilization and included a video demonstrating the effects of focusing the pulses onto a brick.

## Electronic supplementary material

Below is the link to the electronic supplementary material.

Supplementary Material 1

## Data Availability

The datasets generated during and/or analyzed during the current study are available from the corresponding author on reasonable request.
